# Comparación de tres métodos para la medida de la concentración de anticuerpos anti-receptor de tirotropina (TRAb) en el diagnóstico de la enfermedad de Graves. Validación clínica

**DOI:** 10.1515/almed-2020-0053

**Published:** 2020-10-30

**Authors:** Ramona A. Silvestre, Alejandro Almería Lafuente, Lucía Jiménez-Mendiguchía, Ana García-Cano, Rubén Romero López, Belén García-Izquierdo, Cristina Pardo de Santayana, Pedro Iglesias, Juan J. Díez, Ignacio Arribas Gómez, Francisco A. Bernabeu-Andreu

**Affiliations:** Servicio de Bioquímica y Análisis Clínicos, Hospital Universitario Puerta de Hierro Majadahonda, Madrid, España; Servicio de Endocrinología y Nutrición, Hospital Universitario Puerta de Hierro Majadahonda, Madrid, España; Servicio de Bioquímica Clínica, Hospital Universitario Ramón y Cajal, Madrid, España

**Keywords:** anticuerpos anti-receptores de tirotropina, enfermedad de Graves, inmunofluorescencia, quimioluminiscencia, TRAb, validación clínica

## Abstract

**Objetivos:**

La enfermedad de Graves (EG) es una enfermedad secundaria a la presencia de autoanticuerpos anti-receptor de TSH (TRAb) que estimulan la producción de hormonas tiroideas. La medida de la concentración de TRAb es crucial para su diagnóstico etiológico. Los objetivos de este estudio fueron 1. Comparar dos métodos de medida de TRAb por quimioluminiscencia con el método habitual del laboratorio (TRACE-inmunofluorescencia). 2. Determinar la validez diagnóstica de los tres métodos.

**Métodos:**

Estudio retrospectivo de 194 pacientes con petición de TRAb, analizado por: inmunofluorescencia (Kryptor, ThermoFisher) y quimioluminiscencia (Immulite, Siemens y Maglumi, Snibe). Validación clínica: revisión de historias clínicas y clasificación según función tiroidea. Análisis estadísticos: Variables cuantitativas, coeficiente de correlación intraclase (CCI) y gráfica de Bland-Altman, diferencia de medias (Dm). Variables cualitativas, dicotomizadas según puntos de corte, coeficiente Kappa. Correlación: Pearson y regresión de Passing-Bablok. Se estudió la validez diagnóstica de los tres métodos.

**Resultados:**

Kryptor-Immulite: Dm: 1,2 (IC95%: –16 a+18). Passing-Bablok: Error constante (IC95%: –0,8349 a–0,5987). Error proporcional (IC95%: 0,7862–1,0387). CCI: 0,86 (IC95%: 0,82–0,89). Coeficiente-Kappa: 0,68 (IC95%: 0,59–0,78). Kryptor-Maglumi: Dm: −0,3 (IC95%: −12 a+12). Passing-Bablok: Error constante (IC95%): −0,7701 a+0,1621. Error proporcional (IC95%): 0,8571 a 1,3179. CCI: 0,93 (IC95%: 0,89–0,97). Coeficiente-Kappa: 0,53 (IC95%: 0,32–0,74). La enfermedad de Graves se confirmó en 113 pacientes (Kryptor: mejor especificidad y valor predictivo positivo [VPP]. Immulite: mejor sensibilidad y valor predictivo negativo [VPN]).

**Conclusiones:**

Los tres métodos presentan buen rendimiento diagnóstico en la enfermedad de Graves, con resultados superponibles en la intercomparación de Bland-Altman, aunque el estudio de regresión y concordancia muestran no intercambiabilidad y presencia de sesgos.

## Introducción

La enfermedad de Graves (EG), causa más común de hipertiroidismo, es un trastorno autoinmune de la glándula tiroides debido a la presencia de anticuerpos anti-receptor de tirotropina (TRAb). La activación de este receptor por la unión de TRAb puede estimular la producción de hormonas tiroideas e inducir el desarrollo de hipertiroidismo. En este caso, la inhibición por mecanismo de *feed-back* negativo de la secreción hipofisaria de tirotropina (TSH), inducida por las hormonas tiroideas, no se acompaña de una disminución de la síntesis y secreción hormonal, sino que el propio anticuerpo estimula la actividad de la glándula tiroides [1], [2]. Aunque para realizar el diagnóstico de hipertiroidismo es suficiente con los síntomas y signos clínicos y los resultados bioquímicos de TSH, tiroxina libre (T4L) y triyodotironina libre (T3L), también es importante determinar la existencia de un mecanismo inmunológico, en concreto, de los TRAb. Por otra parte, cabe destacar la importancia de los TRAb en el seguimiento de los pacientes con EG y en la predicción de recidivas. La reducción de la concentración de los TRAb o su desaparición ayudan a confirmar la remisión de la enfermedad y sirve para establecer el momento adecuado para la supresión del tratamiento farmacológico. Además, existen estudios en los que el uso de la medida de la concentración de TRAb para el diagnóstico de la EG frente a la gammagrafía tiroidea reduce el coste en un 47% y acelera el diagnóstico en un 46% [3].

En pacientes con EG además de la presencia de TRAb también se pueden detectar otros autoanticuerpos como los anti-tiroglobulina (anti-Tg) y anti-peroxidasa (anti-TPO) [4], [5] y anticuerpos con actividad neutra [6], que introducen un factor que puede complicar la especificidad en el diagnóstico bioquímico de la EG.

Existen diferentes métodos analíticos comerciales para la determinación de TRAb [2], [7], [8]. En algunos pacientes se ha descrito la presencia de anticuerpos que se unen específicamente al receptor de TSH, pero no son capaces de estimular la secreción de hormonas tiroideas, sino que por el contrario, bloquean el receptor [9]. Incluso en otros pacientes se ha descrito la presencia de ambos tipos de anticuerpos [10], [11]. El balance entre la presencia de anticuerpos estimuladores y bloqueadores determina el nivel de función tiroidea, por lo que sería deseable que el método analítico utilizado para medir TRAb fuera capaz de discriminar entre ambos tipos de anticuerpos (bloqueantes y estimulantes) [7], [12].

La activación del receptor de TSH se puede valorar mediante estudios biológicos en los que se mide el aumento del adenosin monofosfato cíclico (AMPc) intracelular consecuente a la activación del adenilato ciclasa a la que está acoplado dicho receptor, metodología que no tiene presencia en los laboratorios clínicos. Una alternativa al modelo biológico sería la aproximación clínica.

En la práctica clínica habitual, en nuestro laboratorio se determina la presencia de TRAb mediante una técnica de inmunofluorescencia basada en la tecnología TRACE (*time-resolved amplified cryptate emission)* (Kryptor, Thermo Fisher Scientific) [13], [14]. Los objetivos del presente estudio son: (i) comparar dos métodos de quimioluminiscencia para la determinación de TRAb frente al método utilizado actualmente en nuestro laboratorio; (ii) determinar la validez diagnóstica de los tres métodos, comparándolos con un *gold standard* basado en criterios clínicos utilizados para el diagnóstico de la EG, ampliamente aceptados [15], [16], [17].

## Materiales y métodos

Se realizó un estudio retrospectivo, observacional y transversal. La recogida de información de las historias clínicas se llevó a cabo en el periodo de tiempo comprendido entre septiembre y diciembre del año 2019.

### Pacientes y muestras

Se incluyeron las muestras de 194 pacientes que llegaron al laboratorio con petición de TRAb entre enero de 2016 y febrero de 2018. Se realizó la revisión de historias clínicas de los pacientes incluidos en el estudio y se clasificó a los pacientes en función de la situación funcional tiroidea: (a) normofunción o eutiroidismo, como situación asociada a concentraciones séricas normales de TSH (0,35–5,0 µUI/mL) y T4 libre (0,7–1,98 ng/dL); b) hipotiroidismo, como concentraciones séricas bajas de T4 libre y elevadas de TSH; y c) hipertiroidismo, como concentraciones séricas bajas de TSH y elevadas de T4 libre y/o T3 libre (2,3–4,2 pg/mL); d) hipotiroidismo subclínico (concentraciones séricas altas de TSH y T4 libre normal y e) hipertiroidismo subclínico (concentraciones séricas bajas de TSH y T4 libre y T3 libre normales).

El diagnóstico de la EG se estableció atendiendo a parámetros clínicos (signos y síntomas de hipertiroidismo, presencia de oftalmopatía, presencia de mixedema pretibial), estudios de imagen (ecografía y gammagrafía tiroideas) y analíticos (hipertiroidismo franco o subclínico con autoinmunidad tiroidea positiva) [16]. Estos criterios constituyen el *gold standard* del diagnóstico de la EG [16], [17]. En caso de discrepancia entre los resultados de la gammagrafía (o la ecografía) y los anticuerpos, la presencia de un bocio difuso hipercaptante en el estudio gammagráfico asociado a hiperfunción tiroidea fue la prueba determinante para establecer el diagnóstico, incluso en aquellos casos de TRAb negativa, circunstancia que puede aparecer excepcionalmente en los pacientes con EG [18], [19]. Así mismo, se consideraron otras variables clínicas para establecer el diagnóstico de EG, como el tiempo de evolución de la enfermedad, el sexo y edad del paciente, la asociación con otras enfermedades autoinmunes, la respuesta terapéutica a largo plazo a los antitiroideos o la presencia de bocio difuso a la palpación.

Las muestras de sangre se centrifugaron a 1.800 × *g* y se separó el suero. Los sueros fueron congelados a −80 °C hasta el momento de la medición. El estudio fue aprobado por el comité ético de investigación clínica (CEIC) del Hospital Universitario Puerta de Hierro Majadahonda.

Se realizó la medición de la concentración de TRAb por tres métodos: el utilizado actualmente en nuestro laboratorio (inmunofluorescencia, Kryptor Compact Plus, ThermoFisher, en adelante Kryptor) y otros dos métodos que emplean la quimioluminiscencia, en concreto en el analizador Immulite 2000 (Siemens Healthineers) (en adelante Immulite) y en el analizador Maglumi 800 (Snibe) (en adelante Maglumi).

El principio de medición del Kryptor se basa en la tecnología TRACE™, que mide la señal emitida por un inmunocomplejo con retardo de tiempo. La base de esta tecnología es la transferencia de energía no radiante desde un donante (criptato de europio) hasta un aceptor (XL665).

Según datos aportados por los fabricantes, en el caso del Kryptor, el límite de detección es 0,27 UI/L. La sensibilidad es 0,82 UI/L y el límite de cuantificación es 0,89 UI/L. Los coeficientes de variación (CV) intraanalíticos son <10% para concentraciones mayores de 1,2 UI/L y <12% para concentraciones entre 1 y 1,2 UI/L. Los CV interanalíticos son 10% para concentraciones mayores de 2 IU/L y <18% para concentraciones entre 1 y 2 UI/L.

El método de medición del Immulite consiste en un inmunoanálisis quimioluminiscente que emplea un par de quimeras de receptor de TSH recombinante humana. Los límites de detección y de cuantificación para el Immulite, según datos aportados por el fabricante, son 0,06 y 0,10 UI/L, respectivamente.

Finalmente, el tercer método analítico utilizado en el estudio es un inmunoanálisis quimioluminiscente indirecto (CLIA system) no enzimático (Maglumi). Según datos aportados por el fabricante, la sensibilidad es de 2,5 UI/L, los CV intra-ensayo son <8% (concentraciones bajas) y <5% (concentraciones elevadas) y los CV inter-ensayo son <8%.

### Análisis estadístico

El análisis descriptivo de las variables cuantitativas incluyó la media y desviación estándar, para las variables que seguían una distribución normal, y la mediana y el rango intercuartílico en caso contrario.

Para la intercomparación de los diferentes métodos se realizaron los siguientes análisis: coeficiente de correlación intraclase (CCI) y la gráfica de Bland-Altman, para las variables cuantitativas. Para las variables previamente transformadas en cualitativas dicotómicas se empleó el grado de acuerdo mediante el coeficiente Kappa. La dicotomización se hizo aplicando los puntos de corte que aparecen en las especificaciones del fabricante: 1,8 UI/L para Kryptor, 1,5 UI/L para Maglumi y 0,55 UI/L para Immulite. Estas medidas se emplearon para evaluar la concordancia. Además, se utilizaron el coeficiente de correlación de Pearson y la regresión de Passing-Bablok.

Para el análisis de la validez diagnóstica de los diferentes métodos se calcularon la sensibilidad (S) y especificidad (E), los valores predictivos positivo y negativo (VPP, VPN), los cocientes de verosimilitud positivo y negativo (CVP, CVN) y las áreas bajo las curvas ROC (ABC).

Siempre que fue posible, se incluyeron los intervalos de confianza (IC) al 95% en los parámetros estimados. Para el análisis estadístico, se utilizó el programa Medcalc (MedCalc® Version 11.4.2.0).

## Resultados

En la Tabla 1 se muestran los datos descriptivos de la población de estudio (n=194), que estaba constituida por una mayoría de mujeres (n=156). La EG se confirmó en 113 de los 194 sujetos incluidos en el estudio. De los 113 pacientes con EG, en el analizador Kryptor, 86 tuvieron un resultado de TRAb positivo (superior al punto de corte) y, de los 18 eutiroideos con EG, 17 tuvieron un resultado de TRAb positivo. En el resto de los pacientes estudiados la positividad del TRAb fue menor del 1%.

**Tabla 1:** Datos descriptivos de la población de estudio.

	Mujeres		Hombres	
n (%)	156	(80)	38	(20)
Edad (años), x (DE)	49	(16)	52	(21)
Eutiroidismo	69	(35)	15	(8)
Enfermedad de Graves, n (%)	29	(15)	6	(3)
Hipertiroidismo, n (%)	73	(38)	20	(10)
Enfermedad de Graves, n (%)	59	(19)	13	(7)
Bocio multinodular, n (%)	3	(1)	1	(<1)
Adenoma tóxico, n (%)	2	(1)	1	(<1)
Otros, n (%)	9	(5)	5	(2)
Hipotiroidismo, n (%)	14	(7)	3	(1)
Enfermedad de Graves, n (%)	4	(2)	2	(1)
Enfermedad de Hashimoto, n (%)	4	(2)	1	(<1)
Otros, n (%)	6	(3)	0	(0)

% calculado respecto al total (n=194).

De las patologías que aparecen en la Tabla 1, se define bocio multinodular hiperfuncionante como la presencia de enfermedad nodular en la ecografía con nódulos hipercaptantes en la gammagrafía e hipertiroidismo y el adenoma tóxico como la presencia de un nódulo tiroideo con hipercaptación en la gammagrafía, con o sin anulación funcional del resto de la glándula tiroides asociado a hipertiroidismo. En la categoría “Otros” se incluyen tiroiditis silente, tiroiditis subaguda, tiroiditis postparto, tirotoxicosis facticia, tiroiditis crónica autoinmune, hipertiroidismo por alemtuzumab, hipertiroidismo por amiodarona y bocio multinodular tóxico por contrastes yodados.

**Figura 1:**
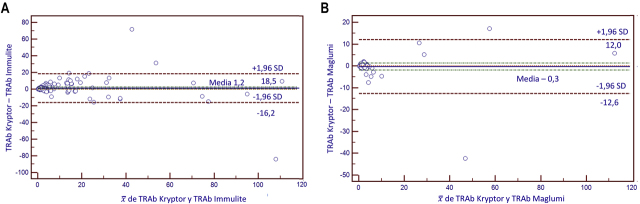
Gráficas de Bland-Altman. (A) Gráfica de Bland-Altman entre los métodos Kryptor e Immulite. (B) Gráfica de Bland-Altman entre los métodos Kryptor y Maglumi.

En la Tabla 2A se muestran los resultados obtenidos para el CCI en los distintos métodos utilizados, que en ambos casos es mayor de 0,8. En la Tabla 2B se muestran los resultados obtenidos de la comparación de las medias de las diferencias frente a la media de los valores obtenidos por cada par de métodos comparados. Estos resultados se pueden ver en forma gráfica en las Figura 1A,B. Las medias están más próximas entre Kryptor y Maglumi que entre Kryptor e Immulite. En la Tabla 2C se muestran los resultados del grado de acuerdo obtenido en la comparación de los resultados obtenidos por los distintos métodos. Previamente, los resultados cuantitativos fueron transformados en cualitativos dicotómicos (positivo/negativo). La comparación de los resultados obtenidos en Kryptor con los de Immulite mostró un coeficiente Kappa ligeramente mejor que el correspondiente a la comparación con Maglumi.

**Tabla 2:** Resultados del análisis de concordancia entre los distintos métodos en la totalidad de los pacientes (n=194).

	CCI	IC 95%
**A**		
Kryptor vs. Immulite	0.8618	0,8229–0,8927
Kryptor vs. Maglumi	0.9343	0,8933–0,9795
**B**	Diferencias $\left(\bar{x}\right)$	IC 95%
Kryptor vs. Immulite	1.2	−16 a+18
Kryptor vs. Maglumi	−0.3	−12 a+12
**C**	Coeficiente Kappa	IC 95%
Kryptor vs. Immulite	0.689	0,592–0,786
Kryptor vs. Maglumi	0.535	0,326–0,745

CCI, coeficiente de correlación intraclase.

En el estudio de comparación de Kryptor frente a Immulite, para determinar la presencia de sesgo, tanto constante como proporcional, se utilizó el diagrama de Bland-Altman, donde se obtuvo una diferencia de las medias (Dm) de 1,2 en valor absoluto.

El estudio de regresión por Passing-Bablok mostró la ecuación: Y=−0,7237+0,9152X, (X=Kryptor, Y=Immulite), con error constante (IC 95%): −0,8349 a −0,5987 y error proporcional (IC 95%): 0,7862 a 1,0387.

En el estudio de comparación de Kryptor frente a Maglumi, el diagrama de Bland-Altman, obtuvo una diferencia de las medias (Dm) de −0,3 en valor absoluto.

El estudio de regresión por Passing-Bablok, mostró la ecuación: Y=−0,1600+1,0218X, (X=Kryptor, Y=Maglumi), con error constante (IC 95%): −0,7701 a 0,1621 y error proporcional (IC 95%): 0,8571 a 1,3179.

Por otra parte, los resultados de los estudios de correlación (Pearson) proporcionaron los siguientes resultados: Kryptor vs Immulite, r=0,78, Kryptor vs. Maglumi, r=0,76.

En la Tabla 3 se muestran los mismos cálculos reflejados en la Tabla 2, pero aplicados al subgrupo de pacientes con EG. El estudio de regresión por Passing-Bablok, para este subgrupo mostró la ecuación: Y=0,3557+1,368X, (X=Kryptor, Y=Immulite), con error constante (IC 95%): 0,035 a 1,368 y error proporcional (IC 95%): 1,117–1,574. El estudio de regresión por Passing-Bablok, mostró la ecuación: Y=0,3913+0,9469X, (X=Kryptor, Y=Maglumi), con error constante (IC 95%): −0,2311 a 1,0073 y error proporcional (IC 95%): 0,5797 a 1,1925.

**Tabla 3:** Resultados del análisis de concordancia entre los distintos métodos en pacientes con enfermedad de Graves (n=113).

	CCI	IC 95%
**A**		
Kryptor vs. Immulite	0,923	0,883–0,948
Kryptor vs. Maglumi	0,982	0,965–0,991
**B**	Diferencias $\left(\bar{x}\right)$	IC 95%
Kryptor vs. Immulite	2,6	−14 a+19
Kryptor vs. Maglumi	0,2	−5,6 a+6,1
**C**	Coeficiente Kappa	IC 95%
Kryptor vs. Immulite	0,586	0,44–0,732
Kryptor vs. Maglumi	0,15	0,11–0,40

CCI, coeficiente de correlación intraclase.

Los resultados del análisis de la validez diagnóstica para la EG de los distintos métodos utilizados en nuestro trabajo se muestran en la Tabla 4. Immulite mostró mayor sensibilidad y mayor VPN que Kryptor y Maglumi. Kryptor mostró mayor especificidad. La VPP fue similar entre Kryptor e Immulite y mayor que Maglumi.

**Tabla 4:** Validez diagnóstica de los distintos métodos.

	Kryptor	(IC 95%)	Immulite	(IC 95%)	Maglumi	(IC 95%)
n	194		194		62	
Sensibilidad %	67	(58–74)	81	(74–88)	58	(42–73)
Especificidad %	80	(69–88)	73	(61–82)	53	(29–75)
VPP %	85	(77–91)	84	(76–90)	73	(56–87)
VPN %	57	(47–66)	69	(58–79)	36	(19–56)
CVP	3.42	(2,14–5,47)	2.99	(2,06–4,34)	1.23	(0,72–2,1)
CVN	0.41	(0,32–0,54)	0.25	(0,17–0,37)	0.8	(0,46–1,38)
ABC	0.81	(0,75–0,86)	0.86	(0,81–0,9)	0.54	(0,45–0,65)

ABC, áreas bajo las curvas ROC; CVN, cociente de verosimilitud negativo; CVP, cociente de verosimilitud positivo; VPN, valor predictivo negativo; VPP, valor predictivo positivo.

Las áreas bajo la curva fueron del mismo orden entre Kryptor e Immulite y menor para Maglumi, siendo los mejores puntos de corte 1,71 UI/L (S=80% y E=82%), 1,42 UI/L (S=73% y E=89%) y 1,48 UI/L (S=61 y E=55%), respectivamente.

## Discusión

El objetivo de este estudio se orientó a comprobar la superponibilidad y a estudiar los indicadores de validez diagnóstica de los diferentes métodos de cuantificación de TRAb, tanto en el conjunto de la población del estudio como en el subgrupo de pacientes con EG.

Se llevó a cabo un análisis de las diferencias medias entre los diferentes procedimientos de medida del TRAb estudiados, así como un estudio de regresión por el clásico método de Passing-Bablok. En cuanto al análisis de las diferencias, los datos se analizaron según el criterio estadístico [20], [21] dada la ausencia de datos publicados en relación con la variabilidad biológica del parámetro en estudio. Según los resultados obtenidos, tanto en el caso del análisis de las diferencias entre Kryptor vs Immulite, como en el caso de Kryptor vs Maglumi, el intervalo de confianza para la media de las diferencias incluye el valor 0, por lo que se considera que no existen diferencias estadísticamente significativas entre los resultados proporcionados por los tres equipos (p≥0,05), por tanto, los resultados serían intercambiables y los tres equipos podrían ser considerados como un equipo virtual único.

Por otro lado, los resultados del estudio de regresión por Passing-Bablok muestran que, en la comparación Kryptor vs. Immulite, el IC 95% de la ordenada en el origen no incluye el 0, por lo que existirían diferencias constantes; el IC 95% de la pendiente incluye el 1, por lo que no existirían diferencias proporcionales entre ambos analizadores. Sin embargo, en el estudio de comparación de Kryptor vs Maglumi, el IC 95% de la ordenada en el origen incluye el 0, por lo que no existirían diferencias constantes; por el contrario, el IC 95% de la pendiente no incluye el 1, por lo que existirían diferencias proporcionales entre ambos analizadores.

En el subgrupo de pacientes con EG la comparación Kryptor vs Immulite muestra error constante y error proporcional, mientras que en la comparación de Kryptor vs Maglumi, no se observa ni error constante ni error proporcional. Hay que destacar que el número de muestras analizadas en Maglumi es inferior a las analizadas por los otros métodos, como se comenta más adelante en las limitaciones del estudio, y como consecuencia el intervalo de confianza de la pendiente es muy amplio, lo que refleja una elevada imprecisión. Por último, la concordancia entre Kryptor y Maglumi mostró un coeficiente Kappa muy bajo.

Hemos obtenido, por tanto, dos tipos de resultados. Por una parte, atendiendo a los datos que resultan de la intercomparación de Bland-Altman se concluye que los tres métodos ofrecen resultados superponibles. También se obtuvieron buenos coeficientes de correlación intraclase, superiores a 0,8. Sin embargo, tanto la inspección visual de los datos pareados y las representaciones gráficas como los resultados de los análisis de regresión por el método de Passing-Bablok, así como la concordancia medida mediante el coeficiente Kappa, hablan en contra de la intercambiabilidad de resultados, detectándose presencia de sesgos. En esta tesitura de resultados divergentes creemos que la decisión más prudente es optar por la no intercambiabilidad de resultados y la conveniencia de aplicar nuevos valores de referencia o nuevos puntos de corte, en el caso de cambiar el procedimiento de medida.

Con respecto a los resultados de la regresión entre Kryptor e Immulite hay que resaltar que la ordenada en el origen es doble en la población total que en el subgrupo de EG, y la pendiente en la población total se acerca más a one que en el subgrupo de EG. Por su parte en este subgrupo la pendiente es mayor (1,368). Una posible explicación es que el reactivo de Immulite solo mida anticuerpos estimulantes [22]. Además, esto justificaría mayor diferencia a valores bajos y que el punto de corte propuesto por el fabricante de Immulite sea mucho más bajo que los otros [14], [22].

En cuanto a la validez diagnóstica cabe destacar que los resultados obtenidos en Kryptor e Immulite son muy semejantes: la sensibilidad y la especificidad son del mismo orden, ligeramente mejor la sensibilidad en Immulite, ligeramente mejor la especificidad en Kryptor. También se obtuvieron resultados similares en estos analizadores en cuanto a la CVP, ligeramente mejor en Kryptor, y el área bajo la curva ROC, ligeramente mejor en Immulite. Sin embargo, Maglumi presenta en los indicadores estudiados unos resultados inferiores a los otros dos equipos en lo que concierne a la determinación de TRAb. Somos conscientes de que el TRAb no es una prueba que se utilice para el diagnóstico clínico de la EG, al menos, en sentido estricto [17], [23], [24], [25], [26]. Es un parámetro que ayuda a establecer el diagnóstico etiológico de la enfermedad. No obstante, además de la intercomparación entre los métodos (objetivo central del trabajo), este estudio nos ha servido para evaluar la capacidad diagnóstica de los diferentes procedimientos de medida estudiados.

Scapatizo et al. [27] encontraron unos buenos resultados de rendimiento diagnóstico para los métodos Kryptor e Immulite en un estudio de 124 pacientes con hipertiroidismo recién diagnosticado y sin comenzar tratamiento. En otro estudio reciente [14] realizado en 383 pacientes (72 con EG, 55 con tiroiditis, 36 con bocio multinodular, 100 con enfermedades autoinmunes extratiroideas y 120 sujetos control) el método Immulite mostró una sensibilidad de 100% y una especificidad de 98%, con un punto de corte de 0,54. Este dato contrasta con el encontrado en nuestro estudio (1,42), muy alejado del citado por Scapatizo et al. [27], a su vez, muy próximo al que propone el fabricante. Además, este método mostró muy buena concordancia con el método de Cobas/Elecsys (Roche) y de TRACK RIA (Brahms, Thermo Fisher). Los resultados de estos trabajos son algo superiores a los encontrados en nuestro estudio, posiblemente porque los pacientes incluidos no habían comenzado el tratamiento para su enfermedad.

Entre las limitaciones del estudio, cabe destacar que la inclusión de muestras de pacientes a los que se solicitó la prueba TRAb puede hacer que se incurra en un posible sesgo de selección, ya que se está seleccionando a pacientes con una mayor sospecha de la enfermedad en estudio. Este criterio de selección podría tener repercusión en la obtención de unos resultados de validez superior o más favorable para los parámetros estudiados.

Otra posible limitación es que se han incluido pacientes en distinto estadio evolutivo y de tratamiento de su enfermedad. En el momento del diagnóstico, todos los pacientes eran hipertiroideos, mientras que durante la evolución de la enfermedad su situación funcional puede cambiar a hipotiroidismo o a normofunción. La variedad de situaciones funcionales debe entenderse en el contexto de un estudio retrospectivo.

Por otra parte, el tamaño muestral representa una fortaleza para los estudios de intercomparación, ya que las guías recomiendan un mínimo de 40, si bien las muestras comparadas con el analizador Maglumi (n=62 para la población completa y n=32 para el subgrupo de EG) fue inferior a las de los otros dos analizadores (n=194). Hay que destacar, por otra parte, que en el estudio de validez diagnóstica se utilizó un *gold standard* robusto y consolidado en la comunidad científica, así como que se aplicó a la totalidad de los pacientes incluidos en el estudio [28].

En conclusión, los tres métodos analizados presentan un buen rendimiento diagnóstico de la EG, siendo Kryptor el que presenta una mejor especificidad y VPP e Immulite el que presenta una mejor sensibilidad y VPN. Los tres métodos ofrecen resultados superponibles en la intercomparación de Bland-Altman, si bien los análisis de regresión por el método de Passing-Bablok y la concordancia medida mediante el coeficiente kappa muestran la no intercambiabilidad de los resultados y la presencia de sesgos.
